# Facilitators and Barriers to Implementing a Local Policy to Reduce Sodium Consumption in the County of Los Angeles Government, California, 2009

**Published:** 2011-02-15

**Authors:** Lauren N. Gase, Tony Kuo, Paul A. Simon, Diane O. Dunet

**Affiliations:** Centers for Disease Control and Prevention; County of Los Angeles Department of Public Health, Los Angeles, California; County of Los Angeles Department of Public Health, Los Angeles, California; Centers for Disease Control and Prevention, Atlanta, Georgia

## Abstract

**Introduction:**

This qualitative study explores facilitators and barriers to a proposed food procurement policy that would require food purchasers, distributors, and vendors of food service in the County of Los Angeles government to meet specified nutrition standards, including limits on sodium content.

**Methods:**

We conducted 30 key informant interviews. Interviewees represented 18 organizations from the County of Los Angeles government departments that purchased, distributed, or sold food; public and private non-County entities that had previously implemented food procurement policies in their organizations; and large organizations that catered food to the County.

**Results:**

Study participants reported 3 key facilitators: their organization's authority to impose nutrition standards, their organization's desire to provide nutritious food, and the opportunity to build on existing nutrition policies. Eight key barriers were identified: 1) unique features among food service settings, 2) costs and unavailability of low-sodium foods, 3) complexity of food service arrangements, 4) lack of consumer demand for low-sodium foods, 5) undesirable taste of low-sodium foods, 6) preference for prepackaged products, 7) lack of knowledge and experience in operationalizing sodium standards, and 8) existing multiyear contracts that are difficult to change. Despite perceived barriers, several participants indicated that their organizations have successfully implemented nutritional standards that include limits on sodium.

**Conclusion:**

Developing or changing policies for procuring food represents a potentially feasible strategy for reducing sodium consumption in food service venues controlled by the County of Los Angeles. The facilitators and barriers identified here can inform the formulation, adoption, implementation, and evaluation of sodium reduction policies in other jurisdictions.

## Introduction

Excess dietary intake of sodium increases blood pressure (1) and can increase the risk of cardiovascular disease, renal disease, gastric cancer, osteoporosis, and left ventricular hypertrophy (2,3). In the United States, the average daily consumption of more than 3,400 mg of sodium greatly exceeds the limit recommended in the 2005 Dietary Guidelines for Americans (2,300 mg for general population and 1,500 mg for blacks, middle-aged and older adults, and those with hypertension) (4,5).

Evidence suggests that reducing the population's intake of sodium can enhance blood pressure control and reduce preventable cardiovascular events (2,6,7). In 2008, a coalition of health organizations and public agencies throughout the United States, led by the New York City Department of Health and Mental Hygiene, engaged leaders of the food industry in an effort to develop a voluntary framework for substantive, gradual reductions over time in the sodium content of many foods (8). Concurrently, several public health departments have expressed interest in local strategies, but local action has been slow. In a February 2010 report, the Institute of Medicine (IOM) recommended that "all state and local health jurisdictions immediately begin to consider developing a portfolio of dietary sodium reduction strategies that make the most sense for early action in their jurisdiction" (9).

In fall 2009, the County of Los Angeles Department of Public Health (DPH) convened a team of experts from its own staff and staff of the Centers for Disease Control and Prevention to identify a feasible strategy to reduce the consumption of sodium in the County of Los Angeles. The team conducted a multistage needs assessment that included an environmental scan of other jurisdictions' actions to reduce sodium consumption, a review of the literature on food environments and nutrition, and development of a logic framework. On the basis of the framework, the team identified several potential policy and environmental change strategies and rated them against a set of criteria. Food procurement policy as a strategy to reduce the intake of sodium among those who eat at food service venues controlled by the County of Los Angeles was deemed most promising for further study. The purpose of this study was to further examine the feasibility of implementing a food procurement policy in the County and, in particular, to identify facilitators and barriers to implementation.

## Methods

The County of Los Angeles is governed by a 5-member board of supervisors. At the time of the study, the board had governmental authority over 37 administrative offices and departments, including the County of Los Angeles DPH. The County is the largest employer in the region, having about 100,000 employees. All design materials and study protocols were reviewed and approved by the institutional review board at the County of Los Angeles DPH before field implementation.

### Definition of a food procurement policy

We defined food procurement policy as an official written policy that would be passed by the County board of supervisors or adopted administratively by a County office or department. Such a policy would require food supplies, as well as meals purchased, distributed, or served by the County's offices or departments, to meet specified nutrition standards, including limits on sodium content. Similar policies were recently enacted in New York City (executive order no. 122, 2008) and in Massachusetts (executive order no. 508, 2009).

In September 2009, DPH and County officials identified key informants, and our team conducted interviews in person or by telephone with 30 key informants representing 18 organizations. There were 3 categories of key informants: 1) 17 representatives from 9 County offices or departments that purchased, distributed, and/or sold food (“departments”); 2) 9 representatives from 5 public and private non-County entities that had previously implemented food procurement policies or nutrition standards in their organizations; and 3) 4 representatives of 4 large organizations that catered food (“food service caterers”) to the County. All key informants contacted agreed to participate in the study.

On the basis of a review of the food policy literature, we developed an interview guide containing 17 or fewer open-ended questions, each having multiple follow-up questions and probes (Table). The questions focused on 4 key dimensions: 1) the work and role of the study participant in the organization; 2) the participant's knowledge and attitudes toward nutrition and sodium; 3) the current nutrition policies in the participant's department or organization; and 4) potential facilitators and barriers to reducing sodium content in food service venues, as perceived by the participant. The specific questions and probes asked of stakeholders varied by the type of organization they represented and their role in the organization. For example, representatives from County departments or offices were asked to react to a hypothetical policy that "strengthened or set nutrition standards, including sodium limits, for the food [their office or department] serves."

Each interview lasted approximately 30 to 90 minutes. One member of our team asked the questions while another took extensive notes. Because many of the interviewees held prominent positions in their organizations and discussions were often about data they considered proprietary or sensitive, most declined to be audiotaped but all agreed that the information could be reported in an aggregate format.

### Analysis

The unit of analysis was the organization (department, non-County entity, or food service caterer). When more than 1 representative from an organization was interviewed, we checked for concordance in their responses. We found some variation in interviewees' perceptions of facilitators and barriers; however, interviewee responses did not deviate regarding their descriptions of organizational operation, procedures, or enforcement of policies. Participant responses were analyzed independently by 2 members of our team, who used the card-sorting method (10) to identify themes relative to facilitators and barriers to policy implementation. At the completion of these analyses, 2 members of our team compared and consolidated themes. A third member was consulted if the first 2 could not reach consensus regarding the themes.

## Results

Results of our key informant interviews are presented as facilitators and barriers to implementing a food procurement policy in the County of Los Angeles. These key themes are ordered according to the frequency they were mentioned by participants (Box).

### Facilitators

**Table:** Box. Facilitators and Barriers to Implementing a Food Procurement Policy to Reduce Sodium, as Reported by Study Participants, County of Los Angeles, 2009

**Facilitators**
**Organizations have the authority to impose nutrition standards** Study participants reported that their departments have the authority to impose nutrition standards, provided they are stricter than current federal or state requirements.
**Serving nutritious food is a high priority** Study participants identified nutrition as a priority. Dietitians employed by county departments expressed willingness to support a policy intended to reduce sodium in foods they served.
**Take advantage of opportunities to build on existing policies** Study participants reported the existence of nutrition policies and standards as a strong foundation on which organizations can build new sodium standards.
**Barriers**
**Food service settings have unique features** Study participants expressed resistance to a one-size-fits-all food procurement policy. They emphasized the need for policies that reflect the unique features of their food service setting, including their existing food standards, other nutritional mandates, the populations they serve, and current contracts.
**Low-sodium foods are costly and may be unavailable** Study participants were concerned about the potential higher costs of healthy, low-sodium foods and the lack of availability of low-sodium items.
**Food service arrangements are complex** Study participants reported that the complexity of their food service arrangements would make it difficult to implement and monitor a food procurement policy that addressed sodium reduction.
**Consumer demand for low-sodium foods is lacking** Study participants raised concerns about the lack of consumer demand for healthy, low-sodium foods, and, correspondingly, were concerned about potential decreases in revenue.
**Taste of low-sodium foods is undesirable** Study participants raised concerns about the undesirable taste of low-sodium foods.
**Vendors prefer to use prepackaged items** Study participants reported relying on prepackaged items, which often contain high amounts of sodium.
**Knowledge and experience in operationalizing sodium standards is lacking** Study participants reported needing additional training and guidance on how to implement sodium standards and reduce the sodium content of the meals they serve.
**Existing contracts are difficult to modify** Study participants reported potential contractual barriers to implementing a food procurement policy, including their inability to change existing contracts and the lengthy process of contract review, which prevented rapid changes to the menu.

**Organizations have the authority to impose nutrition standards.** Study participants from all 9 departments in the County of Los Angeles reported that their respective organizations have the authority to impose nutrition standards, provided they are stricter than federal or state requirements. A food procurement policy passed by the County board of supervisors could have a broad reach, affecting worksite cafeterias, snack shops, and mobile vending trucks; institutions including jails, probation camps, and hospitals; programs for distributing food, such as the meals program for seniors; and County-contracted concessions, such as beach and golf course snack shops. Representatives from each of these settings participated in our study.

**Serving nutritious food is a high priority.** Study participants from 7 of the 9 departments identified nutrition as a high priority; nutrition was of higher importance among the departments that served food to children or seniors than in other departments. Six of the 9 departments employed at least 1 registered dietitian (1 position was vacant). Although the 5 dietitians we interviewed recognized potential barriers to adopting a food procurement policy, reducing sodium was an issue that they thought needed to be addressed. They expressed willingness to support a policy intended to reduce sodium in foods they served.

**Take advantage of opportunities to build on existing policies.** Study participants from 5 of the 9 departments reported that their food service setting had a policy on nutrition or nutritional standards. These departmental representatives reported a high degree of compliance with the required standards on nutrition. However, within the food standards, the standards on sodium were often "recommended" rather than "required" and frequently were not met. Moreover, many nutrition standards were of limited scope, such as those for meals served to clients but not for meals served to staff. Nevertheless, all participants agreed that having existing nutritional policies or standards represented a strong foundation on which their organizations can build new sodium standards.

### Barriers

**Food service settings have unique features.** Study participants from all 9 departments expressed resistance to a one-size-fits-all food procurement policy. They emphasized the need for policies that reflect the unique features of their food service setting, including their existing food standards, other nutritional mandates, the populations they served, and current contracts that cannot be altered. Many participants expressed fear that new nutrition standards applied across all departments would result in their having to serve food that their clients would not eat. Study participants from departments with concession vendors were the most resistant to having one-size-fits-all standards, indicating that such restrictions would prohibit them from selling many of their current items, take away consumer choice, and compromise their department's financial stability. Participants from 3 of the 5 non-County entities confirmed that unique features in their organizations' food environment presented similar barriers to reducing sodium in the different programs, food service units, and concessionaires they oversaw.

**Low-sodium foods are costly and may be unavailable. ** Study participants from all 9 County departments emphasized the need to provide a large volume of food on a small budget and were concerned about the higher costs of healthy, low-sodium foods. Although higher cost was perceived as a barrier by all departments, study participants from non-County entities and food service caterers did not consider this an obstacle. Participants from 4 of the 5 non-County entities, for example, reported that they experienced minimal cost increases after implementing other standards; unfortunately, exact dollar estimates of these changes were not available for examination. Two of these participants said they had been able to negotiate low prices because of the large volume of their purchases. Among the 4 food service caterers, all participants believed that implementing stricter nutrition standards, including lower sodium levels, would not lead to a significant increase in price. All 4 caterers indicated that they cooked their meals from scratch and reported having the ability and the knowledge to decrease sodium in their meals. However, they also said that they have not seen any signal or indication that entities like the County of Los Angeles are interested in purchasing low-sodium products.

Participants from the departments that relied on donations from food or beverage companies raised concerns about their ability to accept donations that did not meet the nutrition standards. However, a participant from 1 non-County entity that relied heavily on donations (53% of all of the food it serves) reported having been able to implement food standards with limits on sodium without decreasing the number of people served. To help meet the standards and contain costs, this entity made changes to its menu, negotiated lower prices on purchased goods, and solicited donations from different companies.

Study participants from several departments and non-County entities described some difficulty in finding a number of low-sodium items, especially products that met multiple standards (eg, standards for fat, calories, and sodium). Participants from 3 departments expressed concerns over whether there were low-sodium foods that could be prepared quickly, as is needed in a concession environment. These participants were also concerned that profit margins might be lower for healthier items. As one concession manager stated, "Cafeterias have to make economic sense in order for vendors to implement a nutritious menu."

**Food service arrangements are complex.** Study participants from 6 of the 9 departments reported that the complexity of their food service arrangements would make it difficult to implement and monitor a food procurement policy that addressed sodium reduction. Many departments reported having to serve meals to multiple clients such as staff and congregate populations and in varying formats such as home-delivered meals, buffet-style meals, and cafeterias that serve food around the clock. Departments also reported having multiple subcontractors or grantees that prepare food differently and have varying levels of experience implementing nutrition guidelines. The complexity of the food service arrangements led these participants to conclude that it would be challenging and time-consuming to monitor adherence to nutritional standards that may involve multiple recipes and menus.

**Consumer demand for low-sodium foods is lacking.** Study participants of 5 of the 9 departments raised concerns about the lack of consumer demand for healthy, low-sodium foods and, correspondingly, were concerned about potential decreases in revenue. Departments that relied on sales to the general public or held contracts with food or beverage companies were particularly concerned. One participant thought that consumer demand drove the food that the concessionaires served, citing the greater presence of healthy foods in concessions in more affluent areas. Study participants from all 5 departments emphasized the need for further public education to raise consumer demand for healthy foods. One participant asked, "If the concessions offer healthy food, what are you going to do to make people buy it? If people don't want the food the concession offers, they go somewhere else."

**Taste of low-sodium foods is undesirable.** Study participants from 5 of the 9 departments raised concerns about the taste of low-sodium foods. All of the food service caterers reported struggling with this issue, emphasizing the need for people to build a "taste profile" for lower-sodium foods, which takes time and can be challenging, especially for groups used to high levels of sodium, such as children. One participant reported that children refused to eat many of the healthy items offered and emphasized the need to consider the culture and upbringing of children in meal planning. To help combat taste barriers, 1 non-County entity used a stair-step approach to gradually reduce sodium content and required that reformulated products pass taste tests. Other departments and food service caterers have also suggested the need to increase access to healthier products by making low-sodium options easily identifiable (through labeling), appealing, and affordable to customers.

**Vendors prefer to use prepackaged items.** Study participants from 4 of the 9 departments reported relying on prepackaged items such as pre-portioned lunch meat and heat-and-serve entrees that often contain high amounts of sodium. Prepackaged items were used by some departments to prevent food-borne illnesses, and other departments lacked access to cooking facilities or had to store products for long periods of time. Participants from all 4 food service caterers described the relative ease of lowering the amount of sodium in products they produce internally compared with prepackaged products they use.

**Knowledge and experience in operationalizing sodium standards is lacking. **Study participants from 3 of the 9 departments reported needing additional training and guidance on how to implement sodium standards and reduce the sodium content of the meals they serve. These participants reported relying on food vendors to supply nutritious food but have not given vendors specific instructions. They expressed a desire for specific guidelines on nutrition that could be understood by local food service facilities and suggested that an approved list of snacks and supplies be provided to vendors. They also suggested providing supplemental training for cooks and staff on how to prepare low-sodium options.

**Existing contracts are difficult to modify.** Study participants from 3 departments reported potential contractual barriers to implementing a food procurement policy, including the inability to change existing contracts and the lengthy process of the contract review. However, study participants from 3 different departments reported some success in working around this barrier and implementing contracts that met specific nutritional requirements.

## Discussion

The County of Los Angeles government has the opportunity to build on its existing infrastructure for providing nutritious meals by incorporating stricter limits on sodium content. Potential facilitators to implementing a food procurement policy include the County's authority to impose nutrition standards, a desire among County departments to serve nutritious food, including dietitians who are ready to help and are already on staff, and existing nutritional policies on which the departments can build.

Consistent with our findings, other research has identified high costs, undesirable taste, and food-preparers' and purchasers' lack of knowledge as barriers to increasing availability of healthy food in other settings (11-13). Although our study participants identified a number of perceived barriers, many departments and non-County entities have already demonstrated the feasibility of implementing nutritional standards that include limits on sodium. For example, despite perceptions of increases in cost, more than one participant reported minimal cost increases after implementation of policies, which they attributed to good skills in negotiating contracts. Building the skills of County purchasers in negotiating contracts may be one way to combat concerns about cost. Additionally, although the perceived undesirable taste of low-sodium foods was often reported as a barrier, some studies suggest that gradual reductions in sodium content might go undetected by the consumer (14,15).

To address the particular needs and constraints of diverse departments within the County of Los Angeles, one option to consider is adoption of department-specific ("venue-based") nutrition standards, having separate guidelines and standards for each food service setting. Developing venue-based standards was recommended by most of the County representatives interviewed. A complementary approach is to phase in nutrition standards gradually. This approach may help to demonstrate to more reluctant departments the feasibility of providing healthy, low-sodium foods in a profitable manner. Other strategies, such as subsidizing the costs of low-sodium options or implementing a public education campaign promoting sodium reduction, can augment these approaches (6,13-15).

Although our study provides qualitative data on the feasibility of local action, it has several limitations. First, although efforts were made to identify all County departments that purchased, served, or sold foods, some may have been missed during the study selection process. Second, it is unclear to what extent the perceived barriers identified by County departments will actually pose a problem if a food procurement policy is adopted. For example, although costs were reported as a barrier by all County departments, the food service vendors interviewed reported that healthy, low-sodium options could be provided for about the same cost as food items currently being served. This claim could not be verified because these food vendors declined to provide actual cost data. Although our study findings are not intended to be generalizable, the facilitators and barriers as well as the lessons learned from this qualitative study may be relevant for other local jurisdictions.

Further research is needed to examine the processes that several jurisdictions have used to successfully implement food procurement policies and to quantify their long-term health and economic effects on the targeted population (eg, level of sodium reduction achieved). County of Los Angeles DPH staff are now investigating the potential costs and benefits of implementing a food procurement policy in the County.

The recent coverage of the National Sodium Reduction Initiative (8) by news media has increased interest in food policies that can potentially reduce sodium consumption in the population. The facilitators and barriers discussed in this study may be useful for other jurisdictions as they consider the feasibility of adopting and implementing food procurement policies as a strategy to reduce to sodium content of the food they serve.

## Figures and Tables

**Table:** Interview Questions Asked of Key Informants, County of Los Angeles, 2009

Category of Question	Key Informant Category		

	County^ a^	Non-County^b^	Caterers^c^
Food service settings	1. What settings or programs or concessions/vending do you have that purchase, distribute, or serve food? 1a. How many meals are you responsible for serving? 1b. Who do you serve (patron profile)? 2. Describe the contract process. (Probes: How many vendors or contracts do you currently have? What factors are important for contractor selection? How often are contracts renegotiated?)	1. What settings or programs or concessions/vending do you have that purchase, distribute, or serve food? 1a. How many meals are you responsible for serving? 1b. Who do you serve (patron profile)? 2. Describe the contract process. (Probes: How many vendors or contracts do you currently have? What factors are important for contractor selection? How often are contracts renegotiated?)	1. With whom do you contract in the County of Los Angeles governmental system? What other types of entities do you contract with? 1a. How many meals are you responsible for serving? 1b. Who do you serve (patron profile)?
Knowledge and attitudes	3. To what extent is the nutrition content of the food you serve a priority? 3a. Are you concerned about the amount of sodium in the foods you offer? Why/why not? 4. Do you think it is feasible to reduce the amount of sodium in the foods you offer? Why/why not?	3. How important do you think it is to implement policies/strategies to reduce sodium consumption in the population?	2. To what extent is the nutrition content of the food you serve a priority? 3. Are you concerned about the amount of sodium in the foods you offer? Why/why not? 4. Do you think it is feasible to reduce the amount of sodium in the foods you offer? Why/why not?
Current nutrition policies	5. Do you currently have nutrition standards or policies aimed to reduce sodium consumption? 5a. What settings do these policies/strategies cover? 5b. Are they voluntary or mandatory? 5c. What has been the level of compliance/ adherence? 5d. What barriers do you/have you faced in implementing these policies or strategies? How have these been overcome?	4. What policies or strategies have you implemented (or "are working to implement") to improve nutrition or to reduce sodium consumption? 4a. Why were these policies/strategies chosen? 4b. Whom do these policies/strategies target? 4c. What has been the level of compliance/adherence? 4d. What has been the impact? 4e. What have been the costs? 4f. What barriers do you/have you faced? How have these been overcome?	5. Do you currently have nutrition standards or policies aimed to reduce sodium consumption? 6. What barriers do you/have you faced? How have these been overcome?
Facilitators and barriers for reducing sodium	6. Where are there opportunities to enhance existing policies or strategies to reduce sodium consumption? 7. How feasible do you think it would be to implement a policy that strengthened or set nutrition standards, including sodium limits, for the food you serve? 7a. How might such a policy affect your organization and your work? 7b. How easy or difficult do you think it would be to manage and enforce this policy? 7c. What barriers might be faced in implementing this policy or strategy? How might these barriers be overcome?	5. Where do you see opportunities to implement new policies or strategies to improve nutrition by reducing sodium consumption in Los Angeles County? 6. How feasible do you think it would be to implement a policy/strategy that strengthened or set nutrition standards, including sodium limits, for food served in Los Angeles County?	7. Imagine a policy has been implemented by the County of Los Angeles that limited the amount of sodium in the food that they purchased. 7a. How would this policy affect your organization and your work? 7b. Would this preclude you from bidding for contracts, if they did not provide additional funds? 7c. Are lower-sodium alternatives or salt substitutes available? 7d. Would low-sodium or salt substitute ingredients cost more? How much more? 7e. Could products be reformulated? What would be the costs to reformulate products? 7f. What would be the cost to implement such a policy? 7g. What other barriers might be faced in implementing this policy or strategy? How might these barriers be overcome?

a Organizations from the County of Los Angeles departments that purchased, distributed, or sold food.

b Public and private non-County entities that had previously implemented food procurement policies in their organizations.

c Large organizations that catered food to the County of Los Angeles.
